# Co-Ni Basic Carbonate Nanowire/Carbon Nanotube Network With High Electrochemical Capacitive Performance via Electrochemical Conversion

**DOI:** 10.3389/fchem.2021.655025

**Published:** 2021-10-21

**Authors:** Furui Tan, Hongyuan Chen, Ronghua Yuan, Xuming Zhang, Deliang Chen

**Affiliations:** ^1^ School of Materials Science and Engineering, Dongguan University of Technology, Dongguan, China; ^2^ Department of Applied Physics, The Hong Kong Polytechnic University, Hongkong, China

**Keywords:** Co-Ni carbonate nanowire, carbon nanotube network, supercapacitor, electrode, electrochemical conversion

## Abstract

In this work, the Co-Ni basic carbonate nanowires were *in-situ* grown on carbon nanotube (CNT) network through a facile chemical bath deposition method, which could be further converted into active hydroxide via cyclic voltammetry strategy. A series of carbonate nanowire/nanotube with different Co/Ni ratio revealed the different growth status of the nanowires on CNT network. The nanostructures of the as-synthesized samples were examined via powder X-ray diffraction (XRD), scanning electron microscopy (SEM), transmission electron microscopy (TEM), X-ray photoelectron spectroscopy (XPS) techniques. The Co/Ni ratio of the carbonate largely affected the size of the nanowires, that the low Co/Ni ratio was beneficial for thin nanowire formation and the nanowires loading on CNT network. Subsequently, the electrochemical performance of the Co-Ni basic hydroxides was studied in a three-electrode test system. The nanowires with low Co/Ni ratio 1/2 can form nanowire array on individual CNTs, which exhibited better electrochemical capacitive performance than the composite network with high Co/Ni ratio nanowires after electrochemical activation. The addition of Co enhanced the rate performance of the hydroxide/CNT, especially improved the long cycle stability largely compared to the rate performance of pure Ni converted hydroxide/CNT composite film reported by our previous research. This result is valuable for the design of inorganic electrochemical active composites based on conductive networks for energy conversion/storage applications.

## Introduction

In recent years, effective energy storage and utilization have attracted much attention for the fast development of electronic devices and the increasing environmental problems (Liu et al., 2010; Zhou et al., 2019a) Among various energy storage strategies, electrochemical energy storage usually plays a key role in the individual electrical and electronic devices with the requirement of stable power supplement (Mathis et al., 2019; Wang et al., 2020) As an important part of electrochemical energy storage device, the electrode should match various requirements for effective energy storage and power supplement, such as high conductivity, high power and energy density, long cycle stability, facile synthesis, high utilization, low cost and environmental friendliness. In different electrochemical energy storage devices, the metallic compounds (usually hydroxide or oxide) with high energy densities and capacities but poor conductivity are used as the electrodes (Nguyen and Montemor 2017; Li et al., 2019) To increase the power density and active the batteries materials, the electrodes with high conductivity are necessary (Chen et al., 2019; Kim and Moon 2020) In commercialized electrodes, the simple mixing of electrochemical active materials and the conductive fillers is a common method. However, the conductive additive unavoidably sacrifices overall energy storage capacity and the mixture with low ratio of conductive fillers could not ensure the stable conductive network in the electrodes, which limits the performance of the electrodes (Farzaneh and Hadi, 2019) To enhance the construction of the conductive network in the electrodes, direct growth of electrochemical active materials on the as-prepared conductive network is an effective approach. (Hosseini and Shahrokhian 2018)

Among many kinds of transition group metal (such as Fe, Co, Ni, V, Mn) oxides/hydroxides, Ni(OH)_2_ and Co(OH)_2_ have been widely explored as electrode materials for supercapacitors due to their high theoretical specific capacities and energy densities originated from their reversible faradic redox reactions, but their low electrical conductivity leads to poor rate characteristic. (Zhu et al., 2013; Hosseini and Roushani 2020; Munde et al., 2020a; Munde et al., 2020b; Hekmat et al., 2020; Shobhnath, et al., 2020) Besides, the pristine grown Ni or Co hydroxide were thick with small specific area, which may reduce their contact area with electrolyte and result in a low parctical capacitance. To solve the problems of these Ni/Co hydroxide using as supercapacitor electrode materials, many conductive substrates were employed to improve the conductivity in the practical capacitance and rate performance. Carbon materials including graphene, carbon nanotubes, carbon fibers, Fullerene C60 and conducting polymers like polyaniline, are usually used as conductive substrates on which metal nanoparticles are anchored and grown (Hadi and Saeed 2018; Hosseini and Shahrokhian 2018; Jokar et al., 2018) Among these materials, carbon nanotube (CNT) could be easily assembled to film and/or paper with open pore structures while keep the large specific area, which is beneficial for the loading of active materials for electrodes of electrochemical energy devices (Chen et al., 2015a; Zhao et al., 2019; Zhou et al, 2019b; Dighole et al., 2020) Till now, various metallic compounds have been effectively grown on individual CNTs, including sulfides, (Hou et al., 2017; Paquin et al., 2015; Yang et al, 2017a) hydroxides(Zhao et al., 2014) and oxides (Cai et al., 2014; Dong et al., 2015) The metallic compounds with different kinds of nanostructures (usually nanosheets) can be easily grown on individual CNTs when the latter is used as powder, and the better contact between CNTs and active materials could still exhibit better performance comparing with those composites prepared by simple mixing. (Raviraj et al., 2020) However, their applied status is still in form of powder rather than films or papers, which limits its further effective use as electrode. How to *in-situ* grow these inorganic active materials on CNT films or papers with high loading mass is still need more efforts. In order to address these issues, researchers have carred out various methods to grow metallic oxides on CNT papers or films, such as chemical bath deposition (Patil et al., 2018; Yusof et al., 2020) and electrochemical deposition. (Chen et al., 2016a; Sun et al., 2018; Yang et al., 2017b) However, these active materials are still not effectively grown on CNT films with high stability and cost effectiveness. In our previous work, the ultra-thin amorphous Ni_2_(OH)_2_CO_3_ nanowire arrays were grown on individial CNTs in CNT paper with large mass loading and then these nanowires were *in-situ* converted into Ni(OH)_2_ nano sheets by electrochemical cyclic reaction. The hybrid CNTs paper/Ni(OH)_2_ shows high specific capacitance up to 1400 F•g^−1^ in the first tens of cycles, but decreased to 1000 F•g^−1^ after 1,000 cycles at 2A•g^−1^, which may lead by the structural instability of pure Ni(OH)_2_. (Chen et al., 2015b)

In this research, to further improve the specific capacity and stability, binary Co-Ni hydroxides have been fabricated as supercapacitor electrode by a two-step procedure. The *in-situ* growth of Co-Ni basic carbonates on as-prepared CNT film is achieved by a modified chemical bath deposition method. Binary Co-Ni carbonates with different Co/Ni ratio were fabricated on CNT paper, which reavealed the diameter of the nanowires was increased with the decreasing Ni content ratio in the basic carbonates, while the Co-Ni basic carbonates Co2Ni with high Ni content could form nanowires with thin diameters. When the decreased carbonate nanostructure size match the diameter of individual CNTs, core-shell structures based on individial CNT backbones can be formed. Compared to the rate performance of pure Ni converted hydroxide/CNT composite film reported by our previous research, the introduce of Co enhanced the rate performance of the hydroxide, and largely improved the long cycle stability. The binary Ni/Co hydroxides revealed better electrochemical energy storage performance because of the synergistic contributions of cobalt ions in the redox reactions improved the single nickel hydroxide. Furthermore, electrochemical activation can largely increase the electrochemical capacitance of the composite films with low Co/Ni ratio, suggesting that the scalable, robust and conductive activated CNT composition may serve as a promising candidate for the electrodes of high-performance electrochemical energy storage devices.

## Methods

### Materials

The CNT papers were prepared by a vacuum filtration method. In a typical process, the pristine CNTs were sheared into CNT cotton by high-speed shearing, and then immersed into the solution of hydrochloric acid (5 mol/L) for 48 h to remove catalyst particles. The purified CNTs were sheared into CNT cotton again and dispersed into deionized water by ultrasonic treatment with the help of Tween-80 (as the dispersant). Then, the dispersed CNT solutions were filtered through a microporous cellulose filter membrane using vacuum filtration and washed by deionized water repeatedly to remove remnant dispersants. After dissolving the cellulose filter membrane by acetone, a freestanding CNT paper with a diameter of 40 mm was obtained. All of the chemical reagents were purchased from Sinopharm Chemical reagent Co., Ltd with analytical reagent grade (AR).

### Synthesis

Ni_x_Co_2-x_(OH)_2_CO_3_ was grown on CNT paper by a modified chemical bath deposition method. Typically, NiCl_2_ and CoCl_2_ were dissolved in 20 ml water with Co/Ni ratio 1/0, 2/1, 1/1 and 1/2, are denoted as Co, Co2Ni, CoNi and CoNi2, respectively. The total concentration of NiCl_2_ and CoCl_2_ were kept at 1 M. Then, a piece of CNT paper (20 mg) was immersed into the solution, respectively. At last, 0.62 g urea was solved into the solution. The mixed solution was put into a glass bottle and its top was screwed. The bottle was put into an oven with the temperature of 80°C for 24 h. After that, the CNT paper was taken out and washed by water. The washed paper was dried at 60°C in air.

### Characterization and Tests

The morphology and microstructure of the samples were systematically investigated by field emission scanning electron microscopy (FE-SEM, Quanta 400 FEG, FEI), high resolution transmission electron microscopy (HRTEM, Tecnai G2 F20S-Twin, FEI), and X-ray diffraction (XRD, D8 Advance Powder X-ray diffractometer, Bruker AXS), X-ray photoelectron spectroscopy (XPS, EscaLab 250Xi). The prepared CNT paper was weighed of 10 mg for each and pressed onto the Ni-foam of 1.0 ⅹ 1.0 cm^2^ as the electrode. Electrochemical experiments were carried out in CHI-660C electrochemical workstation and LANHE CT 2001A electrochemical cell test equipment. A three-electrode system was chosen to test the electrochemical performance of the materials. A platinum wire was used as the counter electrode, and a calomel electrode was used as the reference electrode. A 6 M KOH aqueous solution was chosen as the electrolyte. CV performances were tested in a potential range of 0 V up to 0.8 V under scan rates of 5 mV/s. The cycling stability was tested in LANHE 2001A (5V 50 mA) battery station.

## Results and Discussion

### The Effect of the Different Co/Ni Ratios on the Morphologies of Carbonate Nanowires

**FIGURE 1:**
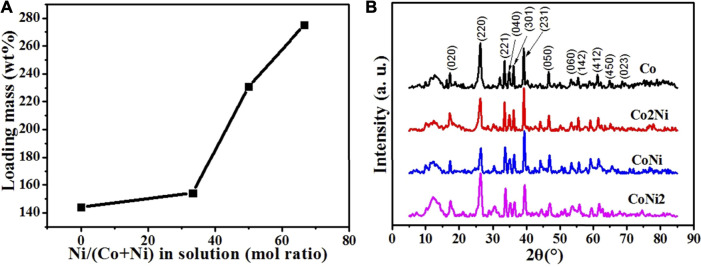
**(A)** The loading mass of Co basic carbonates with different Ni doping degrees. **(B)** XRD patterns of pristine Co/Ni basic carbonate/CNT network.

As shown in Figure 1A, for the carbonate nanowires with Co/Ni ratio 1/0, 2/1, 1/1 and 1/2 denoted as Co, Co2Ni, CoNi and CoNi2, the loading mass of Co/Ni basic carbonate nanowires on CNT network increases along with the decreasing Co/Ni mol ratio. After the deposition of Co/Ni basic carbonate nanowires, the masses of CNT network become 244%, 254%, 331% and 375% of the pristine mass for Co, Co2Ni, CoNi and CoNi2, respectively. It means that the loading ratios of basic carbonate in the composite network are 59, 61, 70 and 73% for Co, Co2Ni, CoNi and CoNi2, respectively. To enhance the performance of the composite network, high loading of active materials is beneficial. However, the formation mechanism of the loading mass under different Co/Ni ratios should be further investigated. The thermal decomposition of Co/Ni carbonate compounds was monitored by TGA, which indicated that Co/Ni carbonate with different Co/Ni ratio exhibit quite similar thermal evolution as shown in Supplementary Figure S1A. As it can be seen in Supplementary Figure S1B, the CoNi2 carbonate sample experience three weight losses : CNT paper caused the first 20% mass loss in the interval [250–550]°C ; a second 12% weigh loss at [550–700]°C is attributed to the decomposition of OH in the composition; a third 6% progressive weight loss at [700–900]°C caused by the degradation of CO_3_.

**FIGURE 2:**
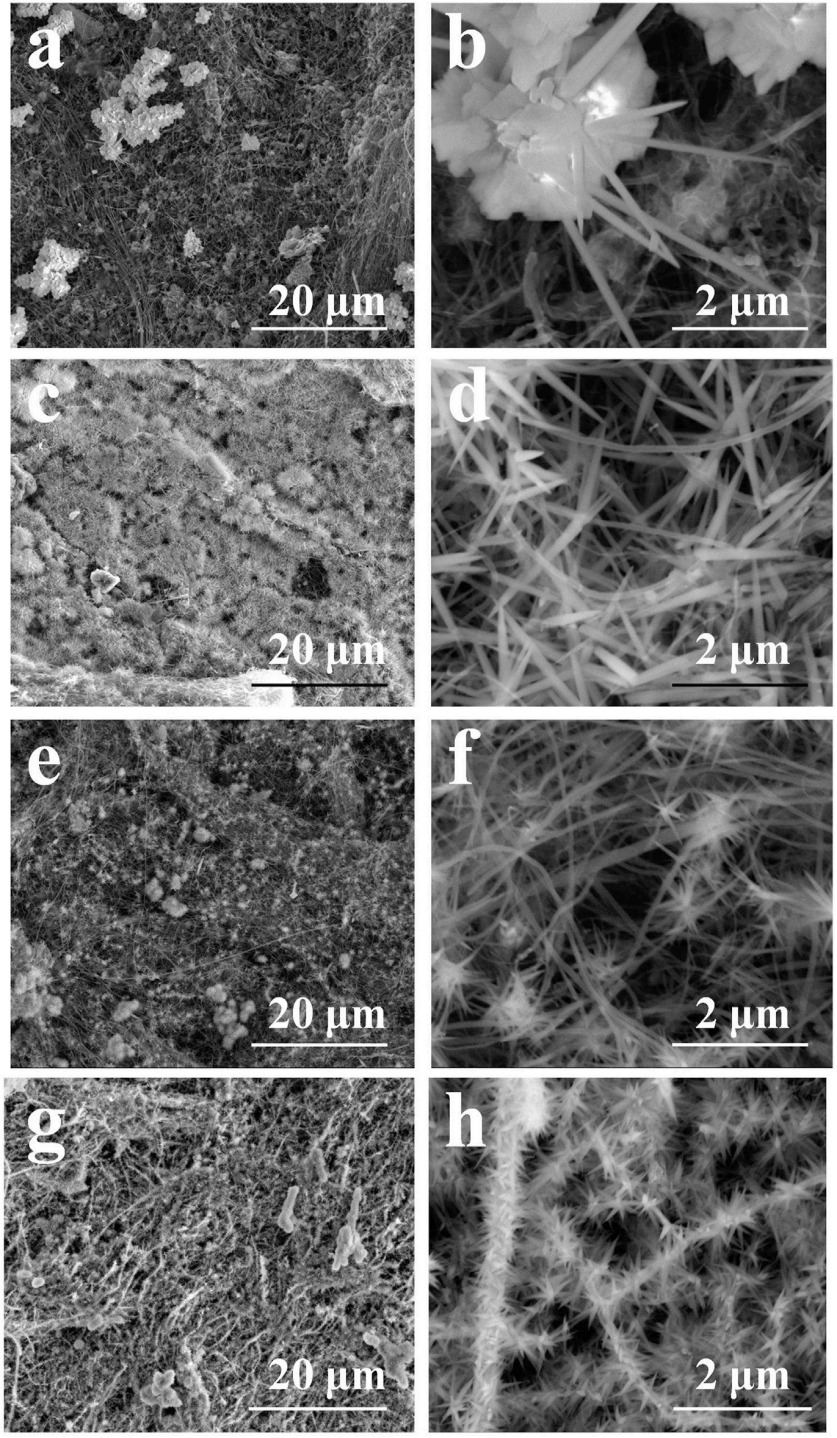
The morphologies of Co/Ni basic carbonate nanowires on CNT network: Co **(A, B)**; Co2Ni **(C, D)**; CoNi **(E, F)**; CoNi2 **(G, H)**.

XRD patterns of the four Co/Ni basic carbonate/CNT composites are shown in Figure 1B. Different from Ni basic carbonate nanowires with typical Ni_2_(OH)_2_CO_3_ structure in our previous discussion,[31] the four samples of Co basic carbonate with Ni doping exhibits typical Co_2_(OH)_2_CO_3_ crystal structure (PDF: 48-0083). It means that even the Ni doping degree is high to 67%, the existence of Co can still keep the crystal structure of the basic carbonate as pure Co basic carbonate. Furthermore, the shapes of Co, Co2Ni, CoNi and CoNi2 are similar as shown in Figure 2, which also indicates the similar crystal structure and consistent with TGA results.

**FIGURE 3:**
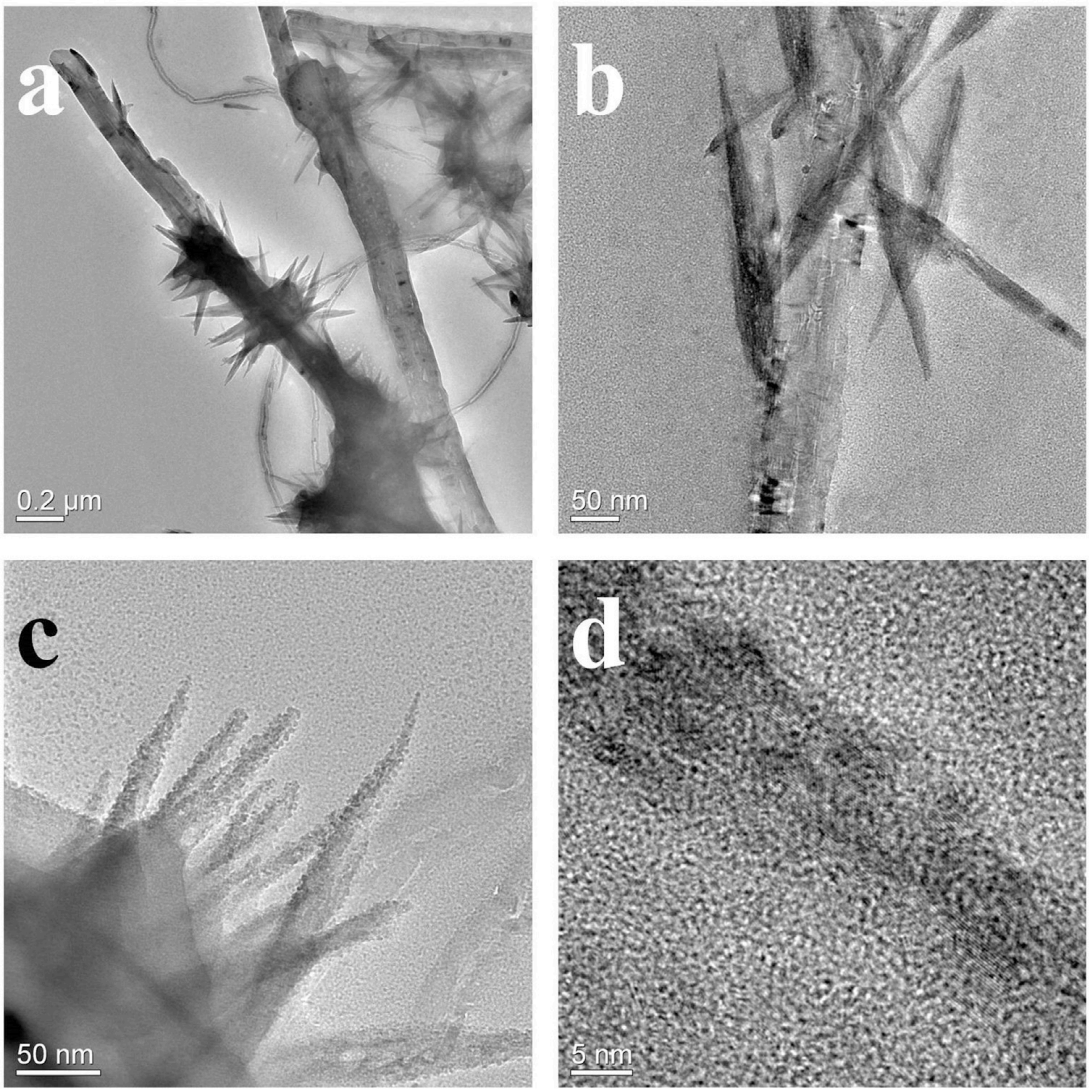
TEM morphologies of CoNi2 basic carbonate nanowire/CNT paper composites **(A, B)** and the CoNi2 basic carbonate nanowire structures **(C, D)**.

According to our previous research, the Ni_2_(OH)_2_CO_3_ formed a nanowire array with thin diameter on individual CNTs (Chen et al., 2015a) Figure 2 shows the micro morphologies of Co/Ni basic carbonates with different Co/Ni ratios on CNT network. The pure Co_2_(OH)_2_CO_3_ nanowire has larger diameter with large particles as the center as shown in Figures 2A,B. Only large particles (1–5 μm) with large-diameter nanowires (100–200 nm) were grown on CNT paper without close contact to individual CNTs on it. When doping the basic carbonate with Ni to achieve Co2Ni, as shown in Figures 2C,D Co2Ni basic carbonate forms dense nanowires on CNT paper, largely different from pure Co basic carbonate with low growth density on CNT paper (see Figure 2A). It means that the main loading form of pure Co basic carbonate on CNT paper may be the particles. However, the diameters of pure Co and Co2Ni basic carbonate nanowires exhibit no obvious difference. The increasing content of Ni will induce thinner basic carbonate nanowire formation as shown in Figures 2E,F (Co/Ni = 1/1). The CoNi nanowire with small diameter can form sea-urchinlike structures on individual CNTs. With higher Ni doping degree, Figures 2G,H reveal that the CoNi2 basic carbonate nanowire forms a mace-like structure on individual CNTs in the network. The nanowires can still be nucleated on CNTs, which should be attributed to their thin diameter. However, the diameter of CoNi2 basic carbonate nanowire is obviously larger than Ni_2_(OH)_2_CO_3_ (Chen et al., 2015b) Generally speaking, the diameter of the Co/Ni basic carbonate nanowires decreases along with the increasing Ni doping, and only high Ni content could ensure the nanowires growth on individual CNTs to form hierarchical core/shell nanowires as shown in Figure 2. TEM images in Figures 3A,B prove that the CoNi2 basic carbonate nanowires are grown on individual CNTs. However, the nanowire is instable under electron beam, and is separated into particles as shown in Figures 3C,D.

### The electrochemical Performance of Co/Ni Basic Carbonate Nanowire/CNT Paper Composite Electrodes With different Co/Ni Ratios

**FIGURE 4:**
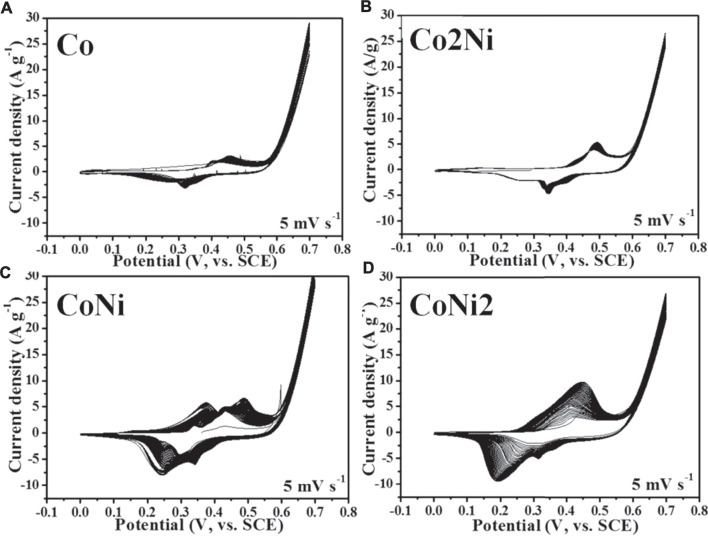
Initial 50 cycles of CV cyclic curves of Co/Ni basic carbonate nanowire/CNT paper composite electrodes with different Co/Ni ratios at 5 mV s^−1^: Co **(A)**; Co2Ni **(B)**; CoNi **(C)**; CoNi2 **(D)**.

Co/Ni basic carbonates can be electrochemically activated from charge/discharge cycles, especially in conductive network such as graphene foam and CNT film. (Chen et al., 2015a; Chen et al., 2016b) The four samples of Co-Ni basic carbonates could be also activated by electrochemical cycles as shown in Figure 4. However, the cyclic voltammetry tests of the four samples exhibit different active extents after 50 cycles, though the loading ratio of basic carbonates in the four composites has no large difference (59% for Co, 61% for Co2Ni, 70% for CoNi, 73% for CoNi2). The Co and Co2Ni with low Ni doping degree have no obvious enlargement after 50 cycles of CV scanning (see Figures 4A,B). However, CoNi2 and CoNi hybrids exhibit effective activation in 50 cycles of CV scanning with enlarged cyclic areas (see Figures 4C,D). This difference cannot be simply explained from the increase of basic carbonate loading on CNT films. It means that the chemical composition or the morphology of the nanowires have large influence on the electrochemical activation of the basic carbonates.

Different from chemical conversion (Zhu et al., 2013) , the electrochemical conversion mechanism can be demonstrated as the equations below, which has also been discussed in our previous reports (Chen et al., 2015a; Chen et al, 2016a). The Ni_x_Co_2-x_(OH)_2_CO_3_ crystal has its pseudocapacitive core Ni^2+^ and Co^2+^, which could be converted to Ni^3+^ and Co^3+^ in electrochemical charge process, and the latter ion exited as Ni_x/2_Co_1-x/2_OOH, which was converted by Ni_x_Co_2-x_(OH)_2_CO_3_. CO^3−^ was resolved into the electrolyte and Ni_x/2_Co_1-x/2_(OH)_2_ kept as a solid on the CNT paper. After that, in the discharge process, Ni_x/2_Co_1-x/2_OOH converted to Ni_x/2_Co_1-x/2_(OH)_2_ and CO_3_^2−^ was not existed in the solid phase on the CNT papers Eqs (1), (2)

$$Ni_{x}Co_{2-x}{\left(OH\right)}_{2}CO_{3}+4OH^{-}\to 2Ni_{x/2}Co_{1-x/2}OOH+CO_{3}^{2-}+2H_{2}O+2e^{-}$$ (1)

$$Ni_{x/2}Co_{1-x/2}OOH+H_{2}O+e^{-}\to Ni_{x/2}Co_{1-x/2}{\left(OH\right)}_{2}+OH^{-}$$ (2)

**FIGURE 5:**
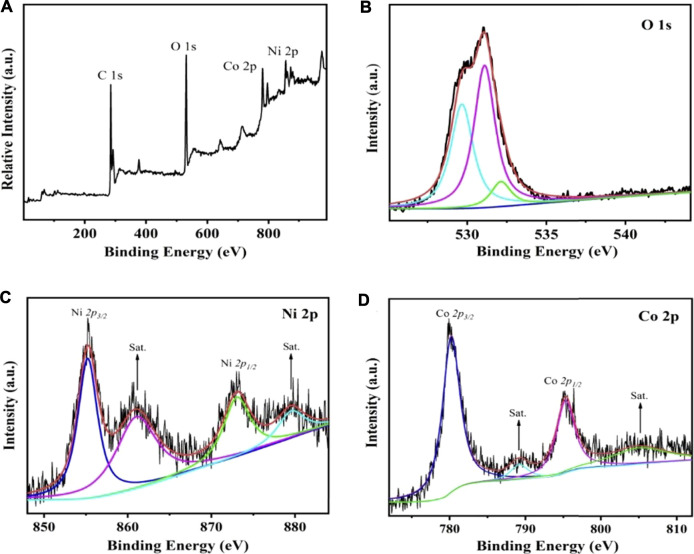
The XPS bands for converted CoNi2 hydroxide/CNT paper composite electrode full serve **(A)** and high resolution data for O 1s **(B)**, Ni 2p **(C)**, Co 2p **(D)**.

X-ray photoelectron spectroscopy (XPS) was utilized to evaluate the surface chemical state of the converted Co/Ni hydroxide materials in the range of 0–1000 eV. As presented in Figure 5A, the diffraction peaks located at 284.3, 529.9, 779.1 and 856.5 eV correspond to C, O, Co and Ni elements in the converted CoNi2 hydroxide materials. The results further indicate that Co/Ni hydroxide were successfully prepared. In the high-resolution O 1 s spectrum in Figure 5B, the peaks at 529.6,531.5 and 532.8 indicate the presence of metal-oxygen bond, O-C-O, and the O-H groups, respectively. The Co 2p and Ni 2p XPS peak spectra were computer fitted using a Gaussian fitting method considering two spin-orbit doublets and two shakeup satellites (marked as “Sat.”). The high-resolution XPS spectrum of Ni 2p (Figure 5C.) reveals that two obvious shakeup satellites (indicated as “Sat”) close to two spin-orbit doublets at 855.7 and 873.1 eV, that can be assigned to Ni 2p_3/2_ and Ni 2p_1/2_ signals, respectively. It suggests the existence of both Ni^2+^ and Ni^3+^. The intense satellite peaks indicate that Ni^3+^ is the majority. In the case of Co 2p XPS spectrum (Figure 5D), the spin-orbit splitting value of Co 2p_1/2_(796.1 eV) and Co 2p_3/2_(781.2 eV) indicated both Co^3+^ and Co^2+^ in the Co/Ni carbonate sample. The weak satellite peaks indicate that the majority of cobalt is Co^3+^. The XPS further demonstrated Ni^2+^ and Co^2+^ could be converted to Ni^3+^ and Co^3+^ in electrochemical charge process, that consistent with our proposed mechanism.

**FIGURE 6:**
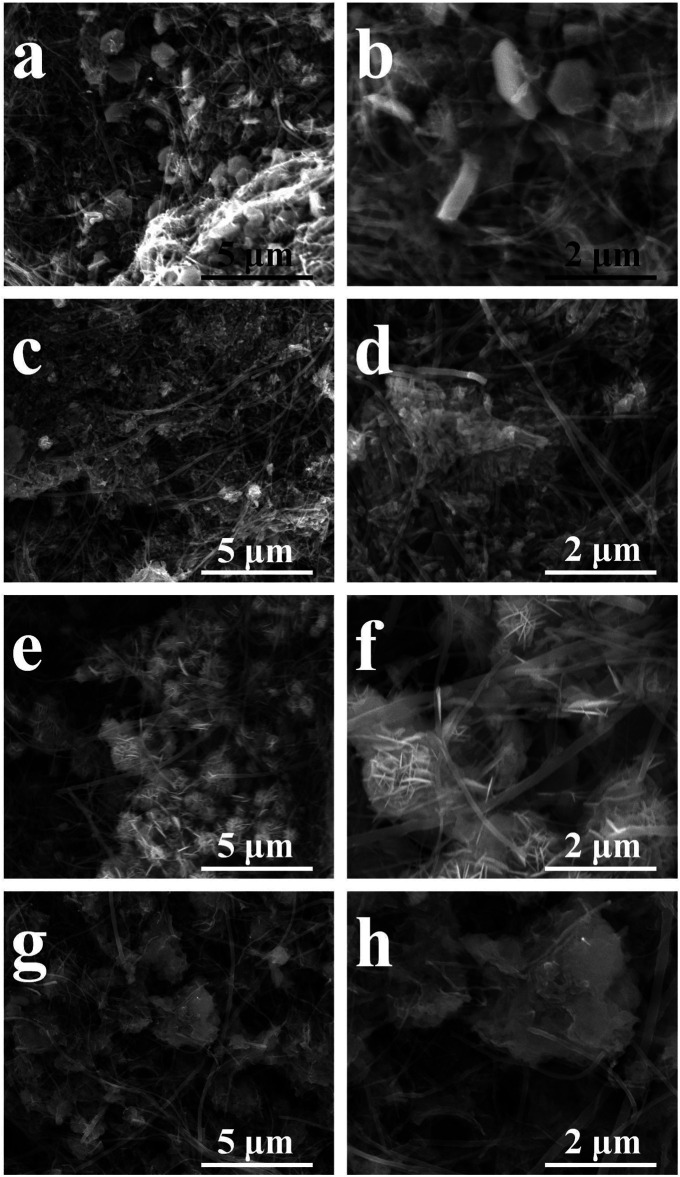
The SEM morphologies of converted Co/Ni hydroxide/CNT paper composite electrodes with different Co/Ni ratios: Co **(A,B)**; Co2Ni **(C,D)**; CoNi **(E,F)**; CoNi2 **(G,H)**.

**FIGURE 7:**
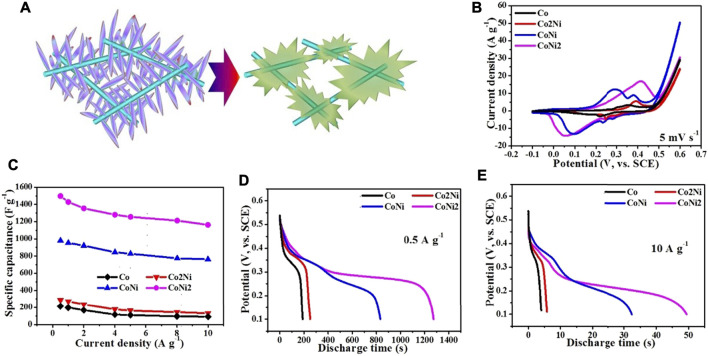
**(A)** Sketch map of the electrochemical conversion from CoNi2 basic carbonate to relative hydroxide; The electrochemical performance of converted Co/Ni hydroxide/CNT paper composite electrodes with different Co/Ni ratios: CV curves at 5 mV s^−1^ **(B)**; rate performance **(C)**; discharging curves at 0.5 A g^−1^ **(D)** and 10 A g^−1^ **(E)**.

The morphologies of the activated composite films are shown in Figure 6. It reveals that the converted hydroxides have different shapes. The pure Co hydroxide has hexagonal shape with large thickness as shown in Figures 6A,B. Furthermore, such nanosheet has no contact with CNT network. For Co2Ni, the converted hydroxide has a particle-like shape and poor contact with CNT network (see Figures 6C,D). Furthermore, CoNi with the increasing Ni doping degree, the converted hydroxide nanosheets form small porous balls as shown in Figures 6E,F, but the thickness of the nanosheets seems still large. At last, CoNi2 hydroxide has thin nanosheet structures with flower-like structures as shown in Figures 6G,H which could largely utilize the active areas of the hydroxides. Figure 7A indicates the electrochemical conversion from CoNi2 basic carbonate to relative hydroxide, that thin nanowires on the CNTs results in tied contact on individual CNTs. Combining the morphologies in Figure 6 and the activation effects in Figure 4, it reveals that the tied contact between Co-Ni and individual CNTs can ensure basic carbonate being converted to thin nanosheets with high electrochemical performance. Although the basic carbonate with low Ni doping degree can also be electrochemically converted to hydroxide, the relative increase of electrochemical performance is not definite.

As shown in Figure 7B, the electrochemical capacitive performance of the converted hydroxides/CNT composites exhibit large difference from the pristine basic carbonates with similar loading ratio. It reveals that the cyclic area of CoNi2 hybrid is larger than other three samples. The area of CoNi hybrid is a little smaller than CoNi2. Two other samples, Co and Co2Ni with low Ni doping degree exhibit much smaller area than the samples of CoNi and CoNi2. This result is consistent with the activation CV curves in Figure 4. From the charge/discharge curves under different current densities in three-electrode system, the rate performance of the four samples can be evaluated and shown in Figure 7C. The introducing of CNT network could effectively ensure the good rate performance of the four samples. All of the four samples have a capacitance decrease of about 20% from current density of 0.5–10 A g^−1^. For the composite film with converted CoNi2 hydroxide, the electrochemical capacitance decreases from 1497 F g^−1^ at 0.5A·g^−1^–1192 F g^−1^ at 10 A g^−1^. For the converted CoNi hybrid, the relative electrochemical capacitances are 921 F g^−1^ at 0.5 A g^−1^ and 762 F g^−1^ at 10 A g^−1^. For the other two samples with low Ni doping degrees in the hydroxide, the practical capacitance of their composites is lower than 300 F g^−1^, which is much lower than the samples with high Ni ratio (Co2Ni and CoNi). It means that high Ni doping degree in Co/Ni basic carbonates can ensure the high practical capacitance of the converted hydroxide. However, comparing with the rate performance of pure Ni converted hydroxide/CNT composite film reported by our previous research, the introduce of Co enhances the rate performance of the hydroxide. For both directly grown Co-Ni hydroxides and converted hydroxides from basic carbonates (Chen et al., 2015b) a core-shell structure with suitable-density arrays of hydroxide/basic carbonate nanosheets or nanowires on individual CNTs in the CNT network usually exhibited higher specific capacitance comparing with other samples. We have summarized electrochemical performance data of related Co/Ni based electrode materials for supercapacitors in Table 1. Even at an ultrahigh current density of 10 A g^−1^, the electrode exhibited a high capacitance of 1192 F g^−1^, which is comparatively higher than those reported earlier in literature using Co/Ni/carbon system (Liu et al., 2020; Hosseini and Shahrokhian 2019; Rahimi et al., 2018; Chen et al., 2015a) It indicates the importance of high-conductive CNT network in the composites. Only close contact between nanostructures with suitable porous structures and individual CNTs can ensure the high electrochemical performance of the composites.

**TABLE 1:** Summarized electrochemical performance data of related Co/Ni hydroxide/CNT paper based electrode materials for supercapacitors.

Composite	Electrolyte	Potential Window (Volts)	Current Density (A g^−1^)	Capacitance (A g^−1^)	Ref.
N-CNTs@Co_2_Ni_1_-LDH	6M KOH	−0.1–0.5	10	340	Liu et al. (2020)
ZnNi_0.5_Co_0.5_Se_2_/Cu_1.8_Se@CC	3M KOH	−0.2–0.6	10	770	Hosseini and Shahrokhian (2019)
Ni-Co-Fe-S@NCAs-NP	3M KOH	−0.2–0.6	10	28	Rahimi et al. (2018)
Ni(OH)_2_CO_3_/MWCNT	6M KOH	−0.1–0.6	10	913	Chen et al. (2015a)
Co/Ni hydroxide/CNT paper	6M KOH	−0.1–0.6	10	1192	This work

Figures 7D,E compare the discharge curve of the four samples under low (0.5 A g^−1^) and high (10 A g^−1^) current densities. The CoNi2, Co2Ni and Co converted hydroxides display the sole discharge plateau potential, but Co/Ni 1/1 converted hydroxide has two plateau potentials, which agrees with two pairs of oxidation/reduction peaks in CV curves (Figure 4 and Figure 7B). It might be attributed to the special oxidation/reduction pair of Co ion in such Co/Ni ration hydroxide. Although Co has more oxidation/reduction pairs than Ni, it cannot be exhibited in many composites. In the CoNi 1/1 hybrid, the combination of Ni and Co with such ratio 1/1 can largely utilize the oxidation/reduction pairs. When increasing or decreasing the Ni ratio in the hydroxide, only one oxidation/reduction pair appears. The full charge/discharge curves of pure Co, Co2Ni, CoNi, CoNi2 hybrids and pure Ni are shown in Supplementary Figure S2. For the CoNi hybrid, which seems a triangle, just similar as electrochemical double-layer capacitor electrodes, shows typical platforms for Ni^2+^/Ni^3+^ conversion. Thus, although the practical capacitance of CoNi hybird is not as high as that of CoNi2 hybrid, it is still valuable to be combined with carbonous materials to assemble asymmetric supercapacitors.

**FIGURE 8:**
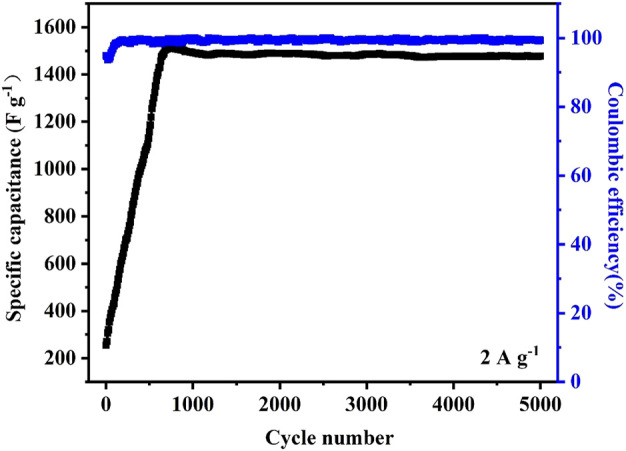
Cyclic performance of CoNi2 hybrid under the current density of 2 A g^−1^.

Figure 8 shows the cyclic performance of pristine CoNi2 carbonate/CNT hybird at the current density of 2 A g^−1^. It reveals a slow activation process comparing with pure Ni carbonate/CNT hybrid. After 700 cycles of charge/discharge, the specific capacitance of the sample increases to a maximum value and still kept around 1600 F g^−1^ after 5,000 cycles with a stable Coulombic efficiency.

## Conclusion

In the present work, for the Co-Ni basic carbonate nanowires grown on CNT network, the size-matching effect is revealed to be a key factor that affects the morphologies and the relative electrochemical capacitive performance of the composites. The basic carbonate with high Ni doping degree will form thin nanowires with array morphology on individual CNTs, which ensures the high performance of the converted hydroxide from basic carbonate and the relative composites. Meanwhile, the addition of Co improved the long cycle stability largely compared to the rate performance of pure Ni converted hydroxide/CNT composite film. This result is valuable for the design of CNT or other nanowires-based network that have electrochemical active materials *in-situ* growth with large loading ratio and high performance.

## Data Availability

The original contributions presented in the study are included in the article/Supplementary Material, further inquiries can be directed to the corresponding authors.
