# Supplementary material for: Co-Ni Basic Carbonate Nanowire/Carbon Nanotube Network With High Electrochemical Capacitive Performance via Electrochemical Conversion
Source: Front Chem. 2021 Oct 21;9:655025. doi: 10.3389/fchem.2021.655025 (PMC8566983; doi:10.3389/fchem.2021.655025)
Supplement: Supplementary file 1 [file DataSheet1.PDF]

Supporting information

Co-Ni basic carbonate nanowire/carbon nanotube network with high electrochemical capacitive performance via electrochemical conversion

Furui Tan[*,1], Hongyuan Chen[1], Ronghua Yuan[1]， Xuming Zhang[2], Deliang Chen[*,1]

[1] School of Materials Science and Engineering, Dongguan University of Technology, Dongguan, P. R. China

[2] Department of Applied Physics, The Hong Kong Polytechnic University, Hongkong, P.R. China.

[*] Corresponding. author: tanfr@dgut.edu.cn; dlchen@dgut.edu.cn (DL Chen)

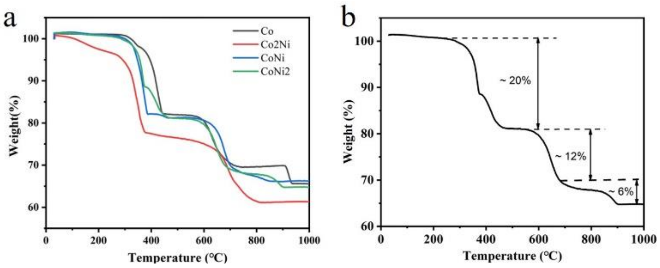

Figure S1. Thermogravimetric Analysis of (a) Co, Co2Ni, CoNi and CoNi2; (b) the thermal evolution stage of CoNi2 carbonate.

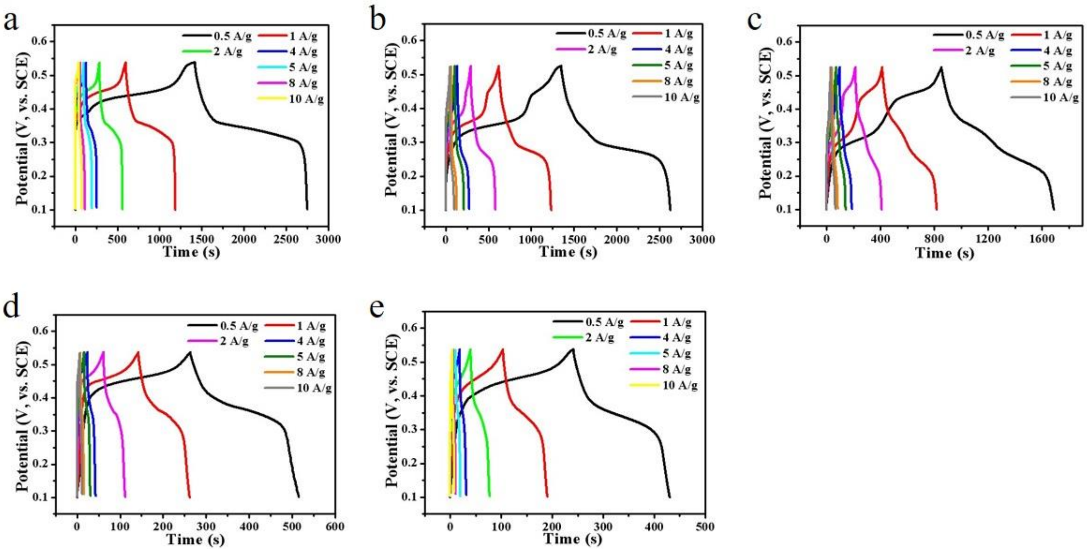

Figure S2. Galvanostatic charge-discharge curve of Ni (a); Ni2Co(b); (c)NiCo; (d) NiCo2; (e) Co.
